# The Nature and Surgical Treatment of Tumours of the Spinal Cord

**Published:** 1896-06-06

**Authors:** E. Percy Paton

**Affiliations:** Surgical Registrar to the Westminster Hospital


### THE NATURE AND SURGICAL TREATMENT OF TUMOURS OF THE SPINAL CORD.

By E. Percy Paton, M.D., M.S., F.R.C.S., Surgical Registrar to the Westminster Hospital.

In a previous article the question of the diagnosis of the existence and the localisation of tumours of the spinal cord has been discussed, and it is now proposed to deal with the nature of the new growths which may be found, the indications for operation, and the method of carrying out operative interference when this has been decided upon.

Of extradural tumours the most common are lipomata, arising from the extradural fat, and sarcomata, which are connected with the bones as a rule, though this is not always the case. Tubercular masses are sometimes found here, but very rarely apart from spinal caries; occasionally also hydatid cysts, enchondromata, and secondary carcinomatous deposits are found in this situation.

The intradural tumours may arise from the membranes or from the cord itself.

In the former case they may be myxomata, fibromata, or sarcomata. When the growth arises from the membranes it is usually not so intimately connected with the substance of the cord itself that its removal involves necessarily any destruction of the nerve fibres of the cord. It must also be borne in mind that syphilitic gummata may also occur here.

Growths involving primarily the cord itself are rarer than those arising in the seats above mentioned. They commonly begin in the periependymal tissue which surrounds the central canal of the cord, and may be syphilitic gummata, gliomata, sarcomata, myxomata, &c.

In the great majority of cases it is clearly impossible before operation to decide what form of growth is present; but it is very important, if possible, to exclude the presence of syphilitic gummata, though to do so is sometimes far from easy. Of course, the first thing is to settle if the patient has had syphilis or not; but, unfortunately, very little weight must be attached to a negative answer to this question. Of course, any sign of the constitutional disease elsewhere is not likely to be overlooked, and especial look-out should be kept for any possibly syphilitic lesion in any other part of the nervous system; rapidity of growth, too, is in favour of syphilis. If, however, any doubt still remains, a course of treatment by means of increasing doses of iodide of potash should be instituted, and the result carefully watched. If, in the course of a month, no improvement takes place, and operation is otherwise indicated, it had better not be delayed longer, unless, indeed, the symptoms have been rather slow in their onset, when the iodide treatment may be tried for rather a longer time.

The points on which most weight should be placed for the diagnosis of tubercular new growth are, the presence in the first place of tubercular lesions elsewhere, then rapid growth at first, with possibly an arrest of progress after a certain amount of advance, is sometimes seen; this does not occur in other forms of tumour, as their progress is one of steady advance. There is usually, also, not much irritation of the cord in tubercular cases, and if there be, it is usually due to inflammation round the growth, after the subsidence of which there may be some recession of symptoms. If syphilis and tubercle can be excluded with a fair degree of certainty, the most probable nature of the growth is sarcoma, and in many cases it is only a high degree of probability in diagnosis that can be reached.

If syphilis has been excluded, the sooner operative measures are resorted to the better, and here the object aimed at is of course a complete cure; but even if this cannot be obtained, relief of pain may be the result of opening the spinal canal in suitable cases. Prolonged delay only results in having ultimately to operate on the patient when in a less favourable condition to bear such interference; it gives a diminished probability of being able to completely remove the growth, and so cure the trouble, and even when it can be removed, restoration of function is less likely, owing to secondary changes which the cord undergoes from prolonged pressure, quite apart from the possibility of its being involved in the growth itself, while if the trouble can be treated by operation, delay allows the patient a prolonged period of unnecessary suffering.

With regard to the operation itself, it need hardly be said that the strictest aseptic precautions must be observed.

The best sort of incision to employ has been much discussed, a U-shaped, Z-shaped, and H-shaped incision having all at different times been recommended. The special advantage of the U-shaped incision is that it permits all the superficial parts to be lifted along with the bones when they have been cut through; then at the close of the operation the flap thus turned back can be replaced. This, however, greatly increases the difficulty of the operation, and it is doubtful if replacing the bones really presents any very great advantages, as without this the space gets subsequently filled in with very tough membrane, and if the periosteum has been preserved with some new bone.

The best incision then is probably a single straight incision over the tips of the vertebral spines. This should be made sufficiently long to allow plenty of room and should be at once carried down to the bone. The thick mass of muscle should then be rapidly cleared from the bones; this is best done rapidly with the knife. The bleeding is rather free, but is venous, and it is best to push on until the soft parts are separated, and then stuff the space with sponges while the same thing is being done on the opposite side, then by waiting a few minutes the packing may be removed without further bleeding. If more room is now needed a transverse incision through the vertebral fascia at either end of the vertical one will allow it to be retracted more freely. The next thing is to divide the bones. It is usually best to snip off the spinous processes with bone forceps first; the laminæ are then partly cut through with a saw set at an angle, and the division completed with bone forceps curved on the flat, so as to enable one to cut without difficulty at the bottom of a rather deep wound. A pair of forceps capable of nibbling away bone is also very useful.

The spinal canal now being open, the condition of the cord can be observed through the dura mater. If it is exposed to very considerable pressure there will be no pulsation in it, which should otherwise be obvious. If the growth be extradural and the right part of the canal has been opened, it will now be exposed, unless, indeed, it be situated on the anterior aspect of the cord which will be indicated by its bulging and want of pulsation. In order to reach it in this situation, more bone will have to be snipped away, and the cord itself made as lax as possible by placing a pillow under the patient above and below the wound, so as to make the spinal column, if possible, concave posteriorly, when the cord may be carefully retracted to one side or the other so as to allow of its anterior aspect being examined. Care must of course be taken of the nerve roots during this manœuvre.

Should it have been necessary, as is probably the case, to divide the peridural fat before thus retracting the cord, this should be done carefully in the middle line, as by this means large veins which give some trouble to control, and which obscure the field of operation when they bleed, are avoided.

Should no new growth be found outside the dura mater, this must now be opened by catching it up in a fine pair of tenaculum forceps and cutting carefully through with a scalpel. It is best to first make a longitudinal incision, and if this does not give sufficient room lateral transverse incisions may be added so as to form a flap, as may be found necessary.

As soon as the dura is punctured there is at once a flow of cerebro-spinal fluid, which must be mopped up carefully with sponges. This flow, however, soon stops if the head of the patient be kept low, and it is important to take this precaution, as otherwise if too much fluid be permitted to drain away convulsions will most probably ensue. The growth must now be carefully dissected away with as little injury as may be to the nervous structures with which it is connected. Unless, however, it arise in the cord itself it has no actual direct connection, as a rule, with these structures. The dura mater must now be closed by fine Chinese twist silk sutures, which may be applied by means of a fine needle on a handle, as in the space at the bottom of the wound there is little room to use the ordinary needles. The wound is now closed in the ordinary way, a drainage tube being put in for twenty-four or forty-eight hours, but not longer, as otherwise there is a tendency to the formation of a track along which cerebro-spinal fluid will continue to escape.

After the dressing to remove the drainage tube, further interference with the wound is unnecessary until it is quite healed, unless, of course, it should fail to run an aseptic course, which becomes a very serious matter, more especially if the meninges have been opened. No splint or jacket is, as a rule, necessary while these cases are still confined to bed, as the articular processes and bodies of the vertebræ are not interfered with, and it is by them that the body weight is transmitted from the upper to the lower segments of the vertebral column.

If the bone removed is replaced the gap of course becomes filled up with bone, but as this is usually not done the space is filled instead with tough fibrous tissue with only a small quantity of bone, when it has been found possible to save some periosteum, from which this new bone is formed. The tough fibrous tissue scar forms, however, a very efficient protection, as was well seen in a recent case in which I had the opportunity of examining the scar nearly a year after I had opened the vertebral canal in a case of spinal caries.

The recovery from paraplegia, &c., is dependent on whether the cord has been too much damaged to recover itself, or whether it has been necessarily damaged during the operative interference, and as it may be some time before the full effect of the operation can be estimated, no hasty opinion of the result should be come to.

When the patient gets up it is advisable at first to let him wear a light protoplastic jacket until the parts around the wound have been thoroughly consolidated, when this support can generally be dispensed with with safety.

